# Computational Modeling of Magnesium Hydroxide Precipitation and Kinetics Parameters Identification

**DOI:** 10.1021/acs.cgd.2c01179

**Published:** 2023-06-23

**Authors:** Antonello Raponi, Salvatore Romano, Giuseppe Battaglia, Antonio Buffo, Marco Vanni, Andrea Cipollina, Daniele Marchisio

**Affiliations:** † Department of Applied Science and Technology, Institute of Chemical Engineering—Politecnico di Torino, Torino 10129, Italy; ‡ Dipartimento di Ingegneria, Università degli Studi di Palermo, viale delle Scienze Ed.6, Palermo 90128, Italy

## Abstract

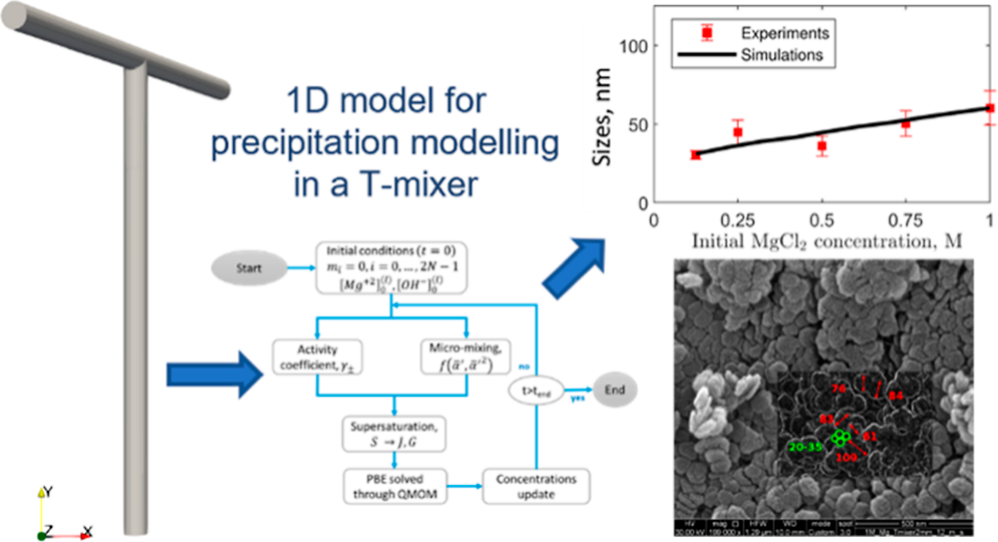

Magnesium is a critical raw material and its recovery as Mg(OH)_2_ from saltwork brines can be realized via precipitation. The effective design, optimization, and scale-up of such a process require the development of a computational model accounting for the effect of fluid dynamics, homogeneous and heterogeneous nucleation, molecular growth, and aggregation. The unknown kinetics parameters are inferred and validated in this work by using experimental data produced with a T_2mm_-mixer and a T_3mm_-mixer, guaranteeing fast and efficient mixing. The flow field in the T-mixers is fully characterized by using the *k*-*ε* turbulence model implemented in the computational fluid dynamics (CFD) code OpenFOAM. The model is based on a simplified plug flow reactor model, instructed by detailed CFD simulations. It incorporates Bromley’s activity coefficient correction and a micro-mixing model for the calculation of the supersaturation ratio. The population balance equation is solved by exploiting the quadrature method of moments, and mass balances are used for updating the reactive ions concentrations, accounting for the precipitated solid. To avoid unphysical results, global constrained optimization is used for kinetics parameters identification, exploiting experimentally measured particle size distribution (PSD). The inferred kinetics set is validated by comparing PSDs at different operative conditions both in the T_2mm_-mixer and the T_3mm_-mixer. The developed computational model, including the kinetics parameters estimated for the first time in this work, will be used for the design of a prototype for the industrial precipitation of Mg(OH)_2_ from saltwork brines in an industrial environment.

## Abstract 2

This work develops a computational model to recover magnesium from saltwork brines through precipitation. Experimental data from two T-mixers are used to infer and validate kinetics parameters. The model aids in designing an industrial prototype for magnesium precipitation.

## 1 Introduction

Magnesium hydroxide is increasingly drawing attention, thanks to the wide range of applications it can be used for. It is particularly appreciated for its environment-friendly applications such as flame-retardant filler^1−4^ or as carbon dioxide absorbent due to its mineral carbonation process.^5^ Moreover, among others, some further important applications are worth to be mentioned: (i) wastewater treatments,^6^ (ii) waste gas treatments such as desulfurization and denitration,^7^ (iii) magnesium oxide synthesis for the use in catalytic industry,^8^ (iv) pharma and nutraceutical industry, (v) refractory industry, and (vi) metallurgical industry as raw material. This wide range of applications is made possible by magnesium hydroxide’s ability to change its physicochemical properties depending on the crystals’ shape and their size distribution. When it comes to satisfying specific requirements, the aim is to synthesize magnesium hydroxide with a crystal size distribution as monodisperse as possible,^9^ the size of which depends on the field of application. As a flame retardant, for instance, hexahedral crystals around 1 μm are required. Conversely, in processes that do not have the production of magnesium hydroxide as their main objective, broader crystal size distribution might influence downstream processes due to inhomogeneous suspension flowability and filterability. Consequently, it is essential to identify precipitation kinetics to control the shape and size distribution of crystals. Several routes are known for magnesium hydroxide synthesis depending on the characteristics required for the final product and briefly described henceforth. Increasing interest arouses the precipitation processes exploiting waste brines and bitterns; in fact, the magnesium concentration in waste brines ranges between 1.1 and 1.7 g/L, reaching a considerably higher value (60 g/L) in bitterns. Therefore, these Mg^2+^-rich streams can react with alkaline solutions leading to the Mg(OH)_2_ precipitation, to be then further separated and collected.

Another route that is widely used in the large-scale process is the hydrothermal method. In this regard, a magnesium precursor, Mg(NO_3_)_2_·6H_2_O for instance, reacts with an alkaline solution in a stirred reactor at room temperature; it is further moved in an autoclave system and hydrothermally treated at constant temperature (150–180 °C), cooled down again at room temperature, separated via centrifuge, washed with water to remove impurities, washed with ethanol to prevent agglomeration and, finally, dried in inert gas atmosphere.^10^ In addition, the solvothermal method is worth to be briefly described; it has many analogies with the hydrothermal method, but it can replace the latter when a high-purity product is needed. In fact, by replacing aqueous solutions with solutions under critical conditions, the final product can reach much higher purities.^11^ These two last processes, however, are expensive in terms of energy required and equipment, so the precipitation process has been preferred as a simpler and cheaper alternative.

In this regard, precipitation tests might be conducted by exploiting different experimental setups depending on the goal to be achieved. For instance, if particle enlargement and high purity are desired, it was shown^12^ that the precipitation process should be performed in a mixed suspension mixed product removal (MSMPR) crystallizer; in this case, no organic additives (commonly used to obtain a micro-sized particle size distribution, PSD) were used. On the other hand, instead, if the crystal sizes required are within the nanometer range, other equipment might be used. In this regard, Higee (high gravity) technologies are employed: (i) spinning disk reactor (SDR), (ii) rotating packed bed (RPB), (iii) T (or Y) mixer reactors. Shen et al.,^13^ for instance, used a novel impinging stream-rotating packed bed (IS-RPB) reactor demonstrating the importance of micro-mixing for synthesizing high-performance nanoparticles. The IS-RPB reactor was primarily used to enhance mass transfer and mixing. In this regard, in the IS-RPB, the reactant streams are fed as jets from opposite directions; this configuration led to reactant direct collision, intensifying micro-mixing within the reactor, and homogeneous distribution in the packed bed. The same concept stands behind the T-mixer choice, in which reactants are fed from opposite sides and collide (and react) within the mixing channel. Schikarski et al.^14^ studied these types of systems finding considerably high mixing efficiencies due to the extremely high turbulence generated. Orlewski and Mazzotti (2020), for instance, used a Y-mixer reactor, to investigate the well-known precipitation process of barium sulfate, both experimentally and computationally.^22^

Instead, the literature review related to magnesium hydroxide has highlighted batch or semi-batch experimental tests only. For instance, Alamdari et al.^15^ investigated magnesium hydroxide precipitation in both configurations to study the precipitation process and infer some kinetics equations. They performed preliminary experimental tests to find operative conditions at which primary nucleation could be neglected. The batch experiments led to rapid generation of supersaturation favoring the formation of many fine particles due to primary nucleation. The semi-batch configuration, instead, led to a coarser product size due to the gradual addition of alkaline reactant within the magnesium precursor solution. Therefore, semi-batch was used, and only secondary nucleation and growth rates were considered, besides aggregation. Moreover, the micro-mixing effect was neglected. Yuan et al.^16^ studied primary nucleation and growth rates within a batch system with low concentrations using the electrical conductivity method (related to ions concentrations). Thanks to this measurement, the induction time of nucleation can be determined, and from this, some kinetic parameters can be inferred.

In this work, we aim at developing a comprehensive model to infer kinetic parameters for primary nucleation, molecular growth, and bridge strength of aggregates. For the sake of clarity, throughout this paper, the following terminology will be used: (i) primary particles to refer to single crystals formed by primary nucleation and enlarged by growth; (ii) aggregation to refer to the formation of primary particles clusters (or secondary particles), where primary particles stick together forming stable bridges due to supersaturation depletion; and (iii) agglomeration to refer to the formation of groups of primary particles and their clusters, which come close to each other and hold that configuration due to weak interaction forces. Eventually, a brief description of the paper structure is provided. The experimental procedure and the main results of experimental tests are described in the first part (Section 2). The model details are explained (Section 3) in the second part. In this regard, attention should be drawn to the identification of the kinetics parameters, because models found in the literature do not account for all the phenomena involved, such as mixing or solution non-ideality. Secondary nucleation is here neglected, because of the high supersaturation and will be studied in further works. Results and conclusions are reported in Sections 4 and 5.

## 2 Experimental Section

### 2.1 Materials and Methods

A purposely made experimental apparatus was built to provide the data for tuning and validating the model and inferring the Mg(OH)_2_ precipitation kinetics parameters. Due to the fast precipitation process, circular cross-sectional T-mixers with a diameter of 2 mm (T_2mm_-mixer) and 3 mm (T_3mm_-mixer) were employed to guarantee rapid mixing of the reactants. The T_2mm_-mixer (T_3mm_-mixer) is composed of two 20 (30) mm long inlet channels merging into a 40 (60) mm long vertical channel, namely, the mixing channel. Magnesium hydroxide precipitation tests were carried out by feeding the T-mixers with magnesium chloride (Sigma-Aldrich) and sodium hydroxide (Honeywell FlukaTM, with an assay >98%) solutions, at variable concentrations and flow rates, according to Table 1. MgCl_2_ and NaOH solutions were prepared by dissolving pellets in ultrapure water. The two solutions were fed to the T-mixers using two gear pumps (Fluid-o-Tech FG series). Eight tests were conducted to allow three effects to be studied: (i) the effect of the concentration (cases from #1 to # 5), (ii) the effect of the flow rate (case from #5 to #7), and (iii) the effect of a change in geometry (case #8). Concerning the effect of the concentration, the chosen flow rate was 1160 mL/min for each inlet solution to have a total flow rate of 2320 mL/min in the mixing channel, as shown in Table 1. For these operative conditions, Battaglia et al.^17^ provided an estimated mixing time of about 2 ms. For a mean fluid velocity of 12.3 m/s, corresponding to the aforementioned flow rate, the effect of the initial MgCl_2_ and NaOH concentrations on the produced Mg(OH)_2_ particles was investigated; MgCl_2_ solutions ranged from 0.125M to 1M (to mimic the magnesium content of real brines) and stoichiometric NaOH solutions were used, as reported in Table 1.

**Table 1:** MgCl_2_ and NaOH Solution Concentrations and Flow Rates Employed in the Experimental Campaign for Precipitation of Mg(OH)_2_ Particles

case	MgCl_2_ [M]	NaOH [M]	mixer diameter (mm)	flow rate in the mixing channel (mL/min)	mean velocity in the mixing channel (m/s)	Reynolds number	estimate mixing time, ms
#1	0.125	0.25	2	2320	12.3	27 251	2.0
#2	0.25	0.5	2	2320	12.3	27 251	2.0
#3	0.5	1	2	2320	12.3	27 251	2.0
#4	0.75	1.5	2	2320	12.3	27 251	2.0
#5	1	2	2	2320	12.3	27 251	2.0
#6	1	2	2	1602	8.5	17 000	2.8
#7	1	2	2	773	4.1	8200	5.9
#8	1	2	3	2714	6.4	19 200	5.6

The effect of reactant concentrations on Mg(OH)_2_ particle sizes formed in the mixing channel was investigated at a Reynolds number of 27 251 (Table 1). The effect of the flow rate was studied by keeping the concentration constant and equal to the highest value (MgCl_2_ 1M, NaOH 2M). Taking case #5 as a reference, the flow rate was decreased by 30% for case #6 and 67% for case #7 resulting in different mixing times (Table 1). A previous publication of ours^17^ gives details of the calculation of mixing times concerning the same operating conditions. Finally, using the T_3mm_-mixer, the effect of changing geometry was studied. To characterize the nanometric size distribution of particles, a Malvern Zetasizer Nano ZSP was used, which is based on the dynamic light scattering (DLS) technique. Before performing DLS analysis, the collected Mg(OH)_2_ suspensions were properly treated: (i) a dilution of the Mg(OH)_2_ suspension was made to reach a particle concentration of 0.3 g/L, (ii) a dispersant poly(acrylic acid, sodium salt) solution was added to reach a dispersant concentration of 4.9 g/kg, namely, about 20 drops of PAA (anti-agglomerant) in 100 mL of diluted suspension, and (iii) the samples were exposed to an ultrasound bath for 5 min. Samples dilution was performed complying with the operating range of the Malvern Zetasizer Nano ZSP. The dispersant was added, and the ultrasound treatment was performed to suppress the agglomeration mechanism. Suspension treatments and DLS analysis were carried out about 2 h after the experimental tests. Particles morphology was investigated to further elucidate the characteristics of the precipitate. Mg(OH)_2_ suspensions collected for Cases #1 and #5 were filtered by employing a Büchner funnel, a Büchner flask, 1.8 μm glass fiber filters (GE Healthcare Life Science Whatman), and a vacuum pump. The obtained cake was washed to remove any reaction by-products (i.e., NaCl), trapped in the cake, then dried in an oven at 105 °C for 24 h and lastly crushed by mortar and pestle. Particles were coated by an extremely thin gold layer (as magnesium hydroxide compound is not conductive) and morphology was assessed by scanning electron microscopy (SEM FEI Quanta 200 FEG).

### 2.2 Experimental Results

**Figure 1:**
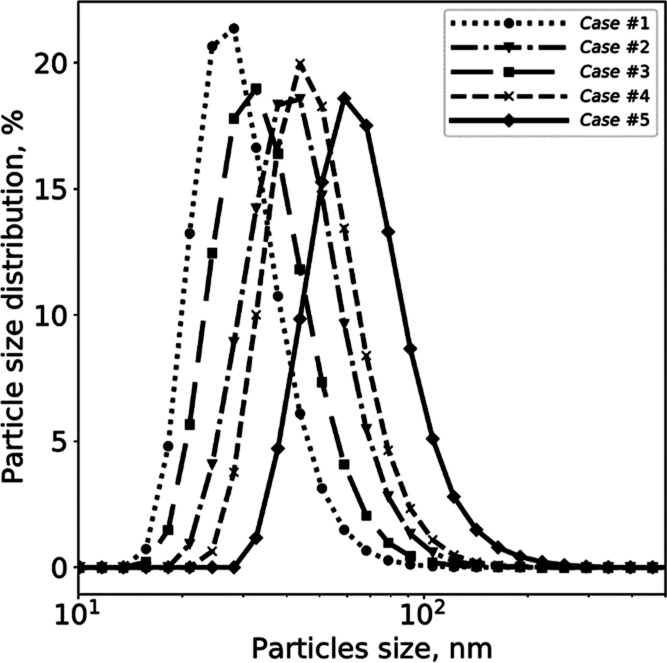
Mg(OH)_2_ PSDs for Cases #1–5. PSDs were obtained after 5 min of ultrasound treatment and using PAA as a dispersant. Measurements were carried out using the Malvern Zetasizer Nano ZSP.

Figure 1 presents the PSDs measured for the first five cases shown in Table 1, referring to the T_2mm_-mixer at a constant flow rate but different initial reactant concentrations. These former five cases were used for model tuning, while the latter three (cases #6–8) were used for validation.

**Figure 2:**
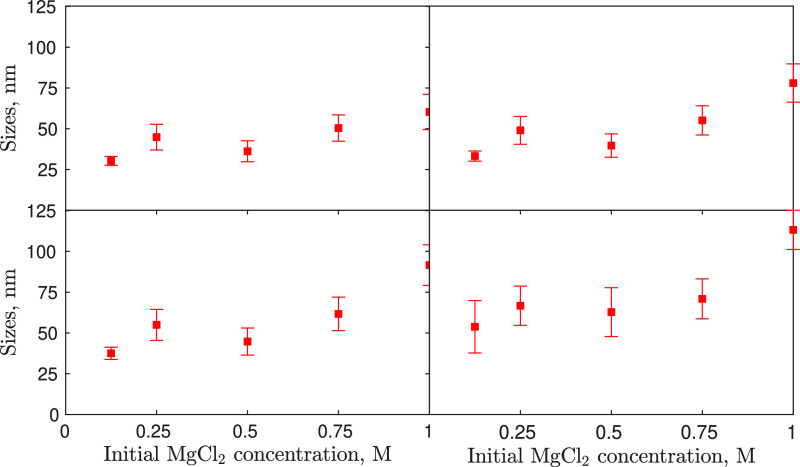
Characteristic sizes from left to right and top to bottom, *d*_10_, *d*_21_, *d*_32_, *d*_43_, derived from PSDs, versus initial MgCl_2_ concentrations (*d*_10_, top-left/*d* top-right/*d*_32_ bottom-left/*d*_43_ bottom-right).

The measured PSDs show an increase in particle size as the initial reactant concentration is increased. A slightly different behavior is observed for Case #3. It is important to highlight, however, that all the experimental tests were affected by errors and uncertainties. Uncertainties might be related to (i) the nanometric nature of the particles, leading to a difficult measurement of their size, (ii) particles dimensions could be affected, although to a small extent, by the fact that they were not analyzed as soon as they were produced, but within 2 h. However, excluding Case #3, a sigmoidal trend has been observed. Characteristic particles sizes were derived from the PSDs and are reported in Figure 2, which reports the *d*_10_, *d*_21_, *d*_32_, and *d*_43_ mean sizes obtained for the five different initial reactant concentrations. These four different mean particle sizes are derived from the PSD by calculating the ratio between the moments of *i*th + 1 and *i*th order of the PSD. The first characteristic size is the *d*_10_ (first order moment/zeroth order moment), namely the number-averaged mean particle size; the second is the *d*_21_ (second order moment/first order moment), namely the length-averaged mean particle size; the third is the *d*_32_ (third order moment/second order moment), namely the surface-averaged mean particle size; and the fourth is the *d*_43_ (fourth order moment/third order moment), namely the volume-averaged mean particle size.

## 3 Computational Modeling

**Figure 3:**
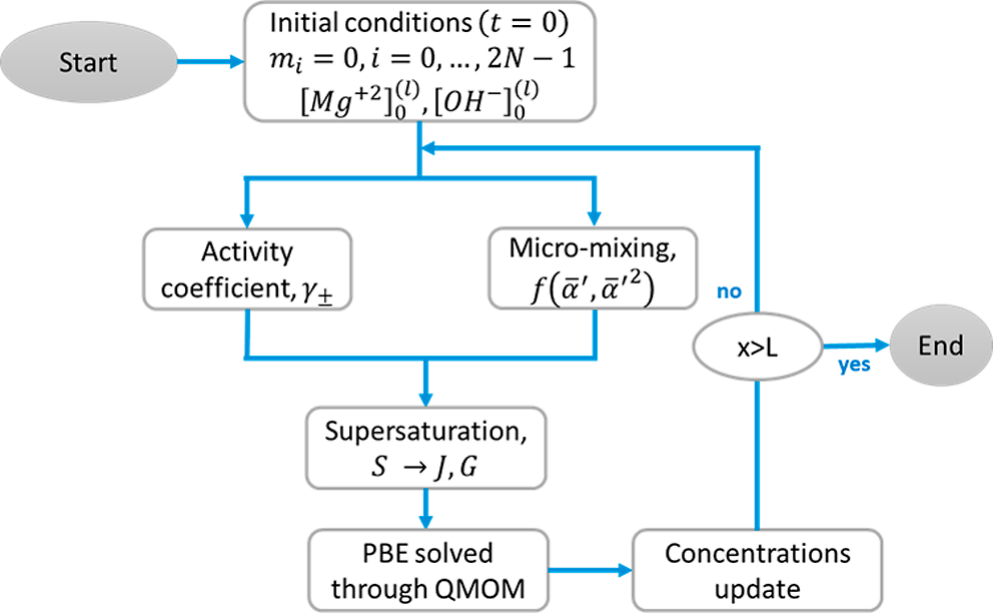
1D model flow chart.

A simplified mono-dimensional model (1D) has been developed and implemented. In this model, several aspects are integrated to accurately reproduce the real physical behavior presented by the experimental evidence: (i) chemical reaction, (ii) solution non-ideality, (iii) particulate processes, and (iv) micro-mixing. The 1D model aims at describing T-mixers, assumed to behave like a plug flow reactor (PFR), but still incorporating information concerning the turbulent fields to account for micro-mixing. It is then employed to determine the Mg(OH)_2_ precipitation kinetics. In Figure 3, the flow chart describing the code implementing the model is presented.

Initially ion concentrations, Mg^2+^ and OH^–^, are set equal to the experimental values at the inlet streams. The moments of the PSD, of order ranging from 0 to 2*N* – 1 (where *N* is the number of nodes used in the quadrature method of moments, QMOM^18^), are set equal to zero because no precipitated solid is initially present. Given these initial conditions, the algorithm evaluates the activity coefficients to be used in the supersaturation profiles and, in parallel, the truly available concentration of Mg^2+^ and OH^–^ that can precipitate due to the chemical reaction between MgCl_2_ and NaOH. Therefore, supersaturation is evaluated. This variable within the model is one of the most important because it represents the driving force, of all the phenomena involved, namely, primary nucleation, growth, and aggregation. More details on the supersaturation variable are provided in Section 3.3. As mentioned, the population balance equation (PBE) is solved in terms of the moments of the PSD by using the QMOM. Once the PBE is solved for the current time step, moments (and their rates) are calculated and used to calculate the precipitated amount of ions from the solution in the solid form. Calculations proceed until the simulation length (input data) is reached. The mono-dimensional model is implemented in MatLab and the ODE integration algorithm used is *ode15s* (also *ode45* was used to check whether the same solution was obtained). This latter was chosen for numerical stability reasons, being the problem stiff. As mixing and reaction times are fast, many physical quantities (particles number, for instance) increase rapidly by several orders of magnitude in a very short time. It is important to emphasize that the mono-dimensional (1D) framework is used to identify precipitation kinetics upon comparison with experimental PSDs through a multivariate constrained optimization routine, as discussed in Section 3.6. One can, therefore, understand the need to use a simplified model that can provide a rapid response, as many function evaluations are necessary. In addition to this fundamental study, the choice of a mono-dimensional model is reinforced by the final application: the design of a prototype for magnesium hydroxide precipitation at the pilot scale. Therefore, a simplified model can be employed to study the influence of operating conditions (e.g., concentration, flow rate) and process parameters (e.g., reaction volume) on the PSDs. As mentioned in the introduction, at the industrial level, specific granulometric characteristics are required depending on the field of application. Having a simplified, computationally cheap tool for numerical investigations allows one to change the input parameters until the desired commercial target is obtained. Once the influence of the parameters on the PSDs has been assessed, a computationally less cheap but physically more complex model can be used for a fine-tuning study. This more complex model can be based, for example, on the idea of solving the PBE (with QMOM) directly within the computational fluid dynamics (CFD) code. With the latter, the influence of flow field gradients (e.g., radial dispersion) can be studied. Once the turbulence is solved, this model can be used in two steps: (i) to assess the mixing of the reactants and, thus, solve the supersaturation field and then (ii) to solve PBE. Performing these two steps consequentially will optimize computing resources. The supersaturation distribution makes it possible to assess, for example, whether radial dispersion is pronounced. If it were, the 1D model could be used for a first qualitative study but it would certainly lead to a quantitative error and the more detailed model should be employed. In conclusion, the associated computational costs are reported. The used computational power refers to a CPU clock frequency of 2300 MHz with 65 Gb RAM. The 1D model is run on a single core, whereas the more complex model is run on multi-cores. The 1D model has an execution time of a few seconds, whereas the supersaturation solution for the more complex solver requires about 9500 times as much (i.e., about 8 h). The solution of the PBE within the CFD code reaches some days of computing.

### 3.1 Computational Fluid Dynamics

**Figure 4:**
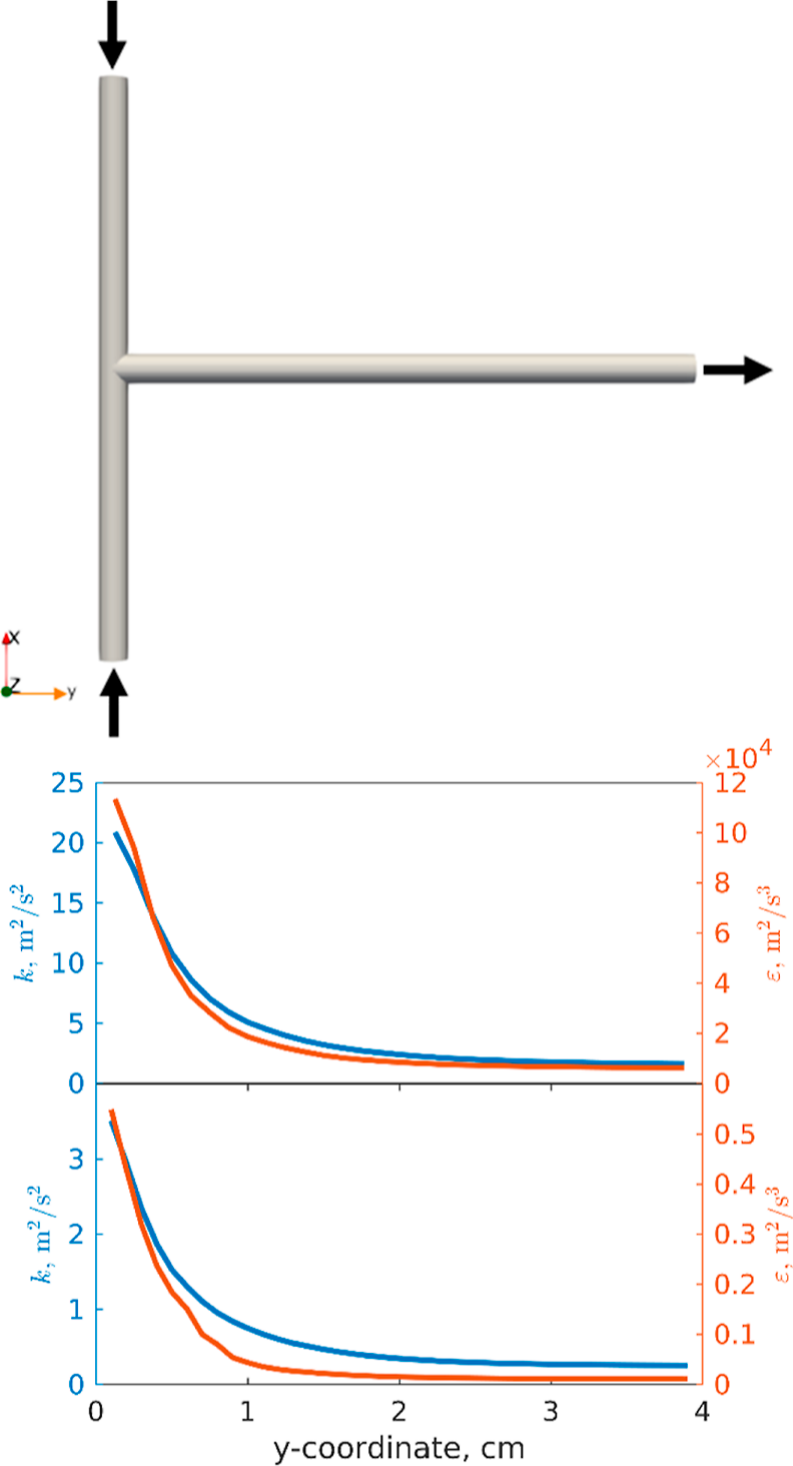
Spatial evolution of TDR, ε, (red line) and kinetic energy, *k*, (blue line) over the mixing channel length. Case study operative conditions: T_2mm_-mixer, 12.3 m/s velocity in the mixing channel namely flow rate of cases #1–5 (top). T_2mm_-mixer, 4.1 m/s velocity in the mixing channel, namely the flow rate of case #7 (bottom).

Since many of the phenomena involved are related to both the turbulent energy dissipation rate (TDR), ε, and the turbulent kinetic energy (TKE), *k*, an accurate description of these quantities is required. Various valid approaches can be used to obtain ε values, such as calculating them using experimental pressure drops (if known) or through CFD simulations. In this work, spatial profiles for the properties of interest were extracted from CFD simulations and employed in the 1D model (see Figure 4). Table 2 provides both the boundary conditions used for the CFD simulation settings and the initial conditions for all the solved fields. In the “Supporting Information,” we also provide additional explanations on why CFD simulations were used in this study.

**Table 2:** Boundary and Initial Conditions (BC-IC) Used in the Simulations

	TKE	TDR	turbulent viscosity	velocity (m/s)
boundary conditions (BC)				
inlet	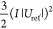	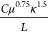	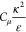	|(6.15 0 0)|
outlet	Neumann condition	Neumann condition	Neumann condition	Neumann condition
walls	kqRWallFunction	epsilonWallFunction	nutkWallFunction	no-slip
initial conditions (IC)				
internal Mesh	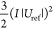	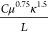	0	(0 0 0)

Where the Neumann condition refers to the gradient of the solved property equal to zero, no-slip refers to the velocity equality between fluid and wall, and the wall functions for the near-wall treatment can be found in the literature.^19^ Simulations were run in OpenFOAM exploiting the *twoLiquidMixingFoam* solver within the Reynold-Averaged Navier–Stokes equation (RANS) approach, computed exploiting the PIMPLE coupling algorithm. Scalable wall functions (already implemented in OpenFOAM) were used for the near-wall treatment, as suggested in the literature.^20^ Eventually, a grid convergence study was performed, resulting in a final grid of about 130 000 cells.

This behavior is in accordance with the known literature. T-mixers develop massive turbulence as described both through experimental tests^21^ and Direct Numerical Simulations (DNS).^14^ NaOH and MgCl_2_ solutions (with two different solution densities and viscosities within the CFD simulations) come from the two inlets impinging along *a* plane, where most of the TKE is transported for convection and dissipated. Keep going along the y-coordinate, namely approaching the outlet, since most of the fluid energy is dissipated, these profiles tend to an asymptotic value. Analogous behavior was found for a similar geometry (Y-mixer) with similar operating conditions.^22^Figure 4 reports the evolution of turbulent profiles for the flow rate of cases #1–5 and #7. It should be noted that the flow rate of case #7 is one-third of the flow rate of cases #1–5. Turbulent properties, however, scale 1 order of magnitude.

### 3.2 Micro-mixing Model and Chemical Reaction

Our study employs a micro-mixing model to account for the molecular-scale mixing of ions required for the formation of Mg(OH)_2_. In very fast processes, micro-mixing can become the rate-determining step. As reported in the “Supporting Information,” accurate predictions of both the trend and experimental data values cannot be achieved without accounting for the micro-mixing. When the micro-mixing model is turned off, predictions for the mean particle sizes are significantly inaccurate and unphysical. Therefore, neglecting micro-mixing would result in an inaccurate description of the experimental data. Micro-mixing is described via the variance of a non-reacting scalar, the mixture fraction, obeying the following ordinary differential equation
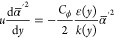
1where *y* is the axial coordinate of the T-mixers and *u* is the average fluid velocity in the axial direction, *C*_ϕ_ was set equal to 2 as reported by Marchisio and Fox (2016).

**Figure 5:**
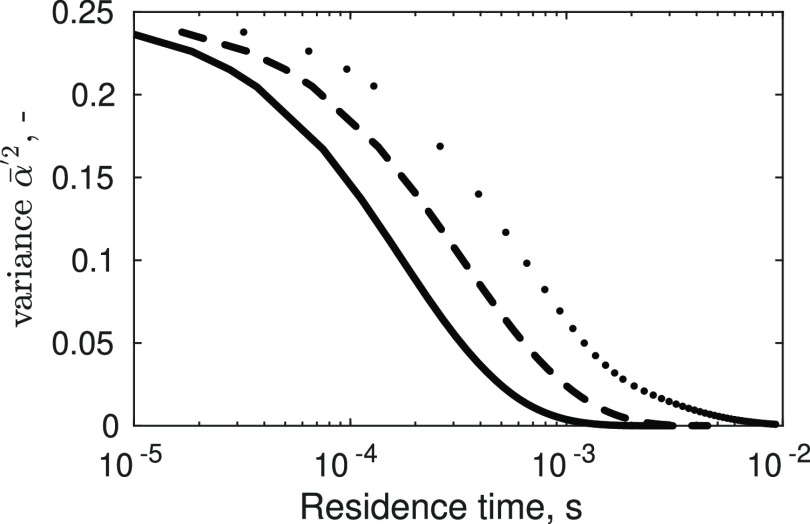
Variance evolution obtained using the TDR and kinetic energy from CFD simulations as a function of the residence time (s) for three flow rates (cases #5–7). The solid line refers to case #5, the dashed line refers to case #6, and the dotted line refers to case #7.

The profiles in Figure 5 are obtained by solving eq 1 by using the turbulent profiles extracted from CFD simulations to estimate the mixing time proportional to the *k*/ε ratio. However, an empirical value for the mixing time, such as the one reported in Table 1, could also be used. In that case, a similar result would have been obtained. The variance evolution, besides the goal for what is used, has important physical implications that can be analyzed; stressing the variance meaning, it tells how fast two reactants can reach the Batchelor (or purely diffusive) scale and, therefore, react. Hence, it is assumed that in the T-mixers, the solution starts with a perfectly micro-segregated condition  for which ions are perfectly macro-mixed  but cannot precipitate (neither nucleate nor grow) because are not available molecularly. To compare variance profiles at different flow rates (Figure 5), the generic y-coordinate within the mixing channel was divided by the velocity corresponding to the investigated flow rate (Table 1) to obtain the profiles as a function of the residence time. All the variance profiles suggest that ions available for the precipitation are micro-mixed within a short period, and this is in accordance with the T-mixers theory.^14^ Furthermore, the effect of flow rates on variance profiles is confirmed quantitatively by empirically estimated mixing times from our previous work^17^ and reported in Table 1. One can note that the time at which variance nulls (Figure 5) is equal to the one experimentally estimated (Table 1). The more the flow rate decreases, the more the turbulence decreases (Figure 4). Therefore, reagents take longer to micro-mix resulting in longer mixing times. The variance decay is used together with the presumed beta probability density function (β-PDF) approach^23^ to evaluate the actual ion concentration available for building up supersaturation, under the assumption of an infinitely fast chemical reaction, as described in the “Supporting Information”.

### 3.3 Calculation of Supersaturation with Activity Coefficients

As mentioned, the computational model accounts for (i) primary nucleation (homogeneous and heterogeneous), (ii) molecular growth, and (iii) aggregation (hydro-dynamic and Brownian). In this regard, a PBE was solved considering all these phenomena, as source terms in the evolution equations for the moments of the PSD. Kinetic parameters related to the source terms were tuned to fit experimentally measured PSDs. The driving force in the precipitation processes is represented by the excess of ions in the liquid compared to the thermodynamic solubility (*k*_sp_) of its solid. Therefore, a dimensionless variable, the supersaturation ratio, or shortly supersaturation, is used to quantify this driving force throughout the process. In our work, supersaturation is defined as follows:
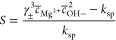
2according to Yuan et al.^16^ where  and  are calculated with the beta-PDF approach (ref., β-PDF approach section) in Supporting Information. In this regard, the hypothesis of instantaneous precipitation reaction was assumed.^24^
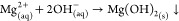
3

**Figure 6:**
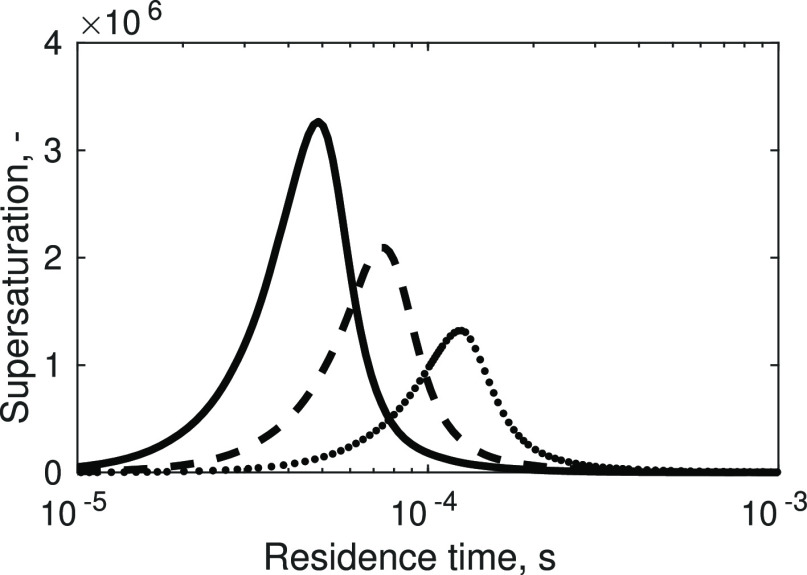
Supersaturation profile reconstructed from the ions concentrations calculated through the model for different flow rates as a function of the residence time (s) for cases #5–7. The solid line refers to case #5, the dashed line refers to case #6, and the dotted line refers to case #7.

It is important to note that these two concentrations are different from the two presented in Section 3.5 that are used for mass balances. The two calculated through the beta-PDF approach and used to calculate the supersaturation, refer to the ones arising after the reaction, see eq 3, and these are subtracted time by time to the total ones, i.e., eqs 16 and 17. Therefore, typical supersaturation profiles reported as a function of the residence time are shown in Figure 6.

This behavior points out that competitive phenomena occur. At the beginning of the process, supersaturation starts increasing due to ions molecular contact (micro-segregation decreases due to high turbulence), and the driving force for precipitation increases. Since supersaturation increases, nucleation and molecular growth occur, resulting in the formation of the precipitate with consequent ions depletion from the liquid phase. The flow rate effect, already introduced with variance profiles, is reinforced by explaining the influence on the supersaturation (Figure 6). By changing the flow rate and, consequently, the turbulence, mixing gets worse and the times within which supersaturation occurs increase (profiles shift to the right, as well as variance). In addition, the maximum local supersaturation value decreases (from solid to dotted line) as the flow rate decreases. Worse mixing produces a lower concentration of molecularly available reagents to react. Lastly, it is necessary to underline the importance of activity coefficients, whose usage is needed to correct the effectively “active” concentration of ions in solution. In this regard, when ion concentration increases, mobility resistance of ions themselves can arise. Counter-ions can form a cloud around co-ions that leads to a shield effect able to reduce their mobility and electrostatic interaction. To consider this effect and to correct analytical concentrations, Bromley activity coefficients for multi-component solutions were used (Bromley 1973). Bromley’s theory is semi-empirical, based on ions electrostatic interactions, and ions were considered: Mg^2+^–OH^–^, Mg^2+^–Cl^–^, Na^+^–OH^–^, and Na^+^–Cl^–^. It is necessary to point out, though, that Bromley’s theory neglects co-ions interactions that may be relevant in some cases. However, since this theory was developed using concentrated seawater as a test solution, Bromley’s theory can be used in our study. This model was implemented for the multicomponent solution because parameters are available in the literature^25^ and each of the presented operative conditions is below 6 M in terms of ionic strength (upper validity limit for Bromley’s theory).

### 3.4 Kinetics Models and Parameters

Primary nucleation (both homogeneous and heterogeneous) was described with the following expression
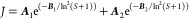
4where ***A***_1_, ***B***_1_, ***A***_2_, and ***B***_2_ are determined by fitting experiments. Molecular growth due to ions’ superficial integration within the growing crystal is described as follows
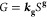
5

where ***k***_**g**_ and **g** are determined again by fitting experiments. Aggregation is considered by accounting for the collision of growing primary particles due to turbulent^26^ and Brownian^27^ fluctuations
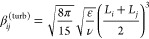
6
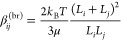
7where *L*_*i*_ and *L*_*j*_ are the sizes of the two colliding particles. Since the two aggregation mechanisms occur in parallel, both kernels are typically summed. Note that, one adjusting coefficient is used because, for the precipitation, the number of particles is extremely high. This means that the number of collisions per unit time [i.e., β_*ij*_^(agg)^] needs to increase.^28^ Moreover, since not all impacts lead to aggregation, Smoluchoski’s collisions theory has been corrected^27^ using aggregation efficiencies or sticking probability. Its mathematical form is different depending on the aggregation mechanisms. The final aggregation kernel used in this work is as follows:
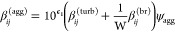
8where
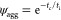
9and *W* is the stability ratio. In our study, because of the zeta potential of the final suspension,^17^ we assume *W* = 1 (electrostatic contribution is favorable for the aggregation due to low repulsive forces, i.e., low zeta potential), while ψ_agg_ is calculated via the interaction time^29^
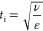
10and the characteristic time required to form a stable bridge between the interacting primary particles, the so-called cementation time
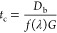
11
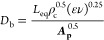
12
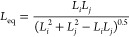
13where ν (10^–6^m^2^/s) is the kinematic viscosity of the liquid phase, ρ_c_ (2.34g/cm^3^) is the crystal density, and ***A***_**p**_ is a tunable parameter related to the bridge strength; *f*(λ) refers to a shape function,^30^ which for spherical particles can be written as follows:
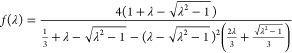
14being λ = *L*_*i*_/*L*_*j*_. The sticking probability is greater than zero (i.e., two particles can aggregate) only if the cementation time is of the same order of magnitude as the interaction time or lower. In other words, two particles could stick together only if the time required for the stable bridge to be formed is at most the one between two rupture events. A key point to consider is the sensitivity of aggregation to the values of the turbulent dissipation rate (TDR), ε.^24^ This is because the turbulent contribution to the aggregation rate is directly related to the ε value (eq 6). Additionally, ε is used to calculate both the interaction time (eq 10) and the cementing time (eq 11), and the ratio of these values allows the aggregation efficiency to be determined. A sensitivity analysis for constant ε values is provided in the “Supporting Information,” and it proves that it is important to carefully evaluate the ε value when modeling the aggregation process.

### 3.5 PBE Solution through QMOM

The integrated modeling approach presented is implemented within the MatLab environment. The PBE is solved by exploiting the QMOM,^18,31^ which for the 1D model is constituted by a system of ordinary differential equations in the following form
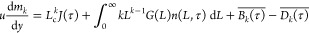
15where *m*_*k*_ (with k = 0, ..., 2*N* – 1) are the moments of the PSD, and the terms on the right-hand side account for primary nucleation, growth, and aggregation. For the simulations, *N* was taken equal to 3 meaning that the evolution of the first six moments was tracked. The system is closed with a mass balance for all reacting ions, i.e., Mg^2+^ and OH^–^^18^
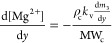
16
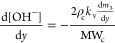
17where ρ_c_ and MW_c_ are the magnesium hydroxide density and molecular mass, *k*_v_ a shape factor assumed equal to π/6 for spheres.

### 3.6 Parameter Identification and Optimization

The optimization routine aims at extracting kinetics parameters for the precipitation process identifying the best fitting between model predictions and collected experimental data. In this regard, a multivariate optimization was performed. Since 8 experimental tests were performed, the dataset was split for tuning and testing the model. Concerning Table 1, cases #1–5 were used for model tuning, and cases #6–8 for validation (Result and Discussion Section). Each experimental test led to a PSD from which four moments ratios were computed. Therefore, the model tuning, which involved 8 unknown parameters, ended up with 20 experimental samples (Figure 2) to be used in the optimization. A target function, exploiting the built model, was used through the *fmincon* MatLab function in which the global error had to be minimized. d⃗ being a vector containing *j* components (i.e., four components because four moment ratios were extracted experimentally) and *i* the index looping on the five investigated concentrations, the global error can be formulated as follows:
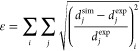
18

Since unphysical sets could arise from optimization, proper parameters range, within the *fmincon* MatLab function, were imposed (see Table 3). These ranges arise from information gathered on similar systems, e.g., barium sulfate.^32^ For example, it is known that these systems have a final suspension density (particles/m3) between 10^17^ and 10^2228^ and these values allow to define a range for the primary homogeneous parameters. The fact that heterogeneous nucleation is typically one (or more) order of magnitude smaller allows to set a range for the corresponding parameters. Experimental measurements^33^ can be used to define a range of the parameter ***B***_1_. The lower bound for the exponent ***g*** for the growth rate was set equal to 1, corresponding to the case of diffusion-controlled growth, whereas the upper limit, 2, was assumed using the theory provided by Mersmann (2001). It is worth mentioning again that if the micro-mixing model is turned off (without changing the number of unknown parameters to be identified), no good fitting is obtained, as described in the “Supporting Information”.

**Table 3:** Ranges for Constrained Optimization Used for Reaching a Local Minimum Satisfying Process Physics and the Lowest Minimum Found

kinetic parameter	units	range for the constrained optimization	value
***A***_1_	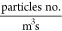	10^19^–10^29^	1.486·10^26^
***B***_1_		250–350	301.44
***A***_2_	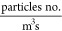	10^10^–10^18^	7.41·10^14^
***B***_2_		10–10^2^	30.34
***k***_**g**_	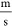	10^–13^-10^–9^	2.51·10^–10^
***g***		1–2	1.001
***c***_1_		0–3	0.835
***A***_**p**_	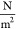	10^0^–10^7^	10^5.897^

## 4 Results and Discussion

Since constrained optimization algorithms generally exploit methods for the local minimum research, different attempts were necessary to land in a local minimum, which could be considered the global one for the actual multi-variable function. In Table 3, the parameters obtained with the best experimental data fitting are reported.

**Figure 7:**
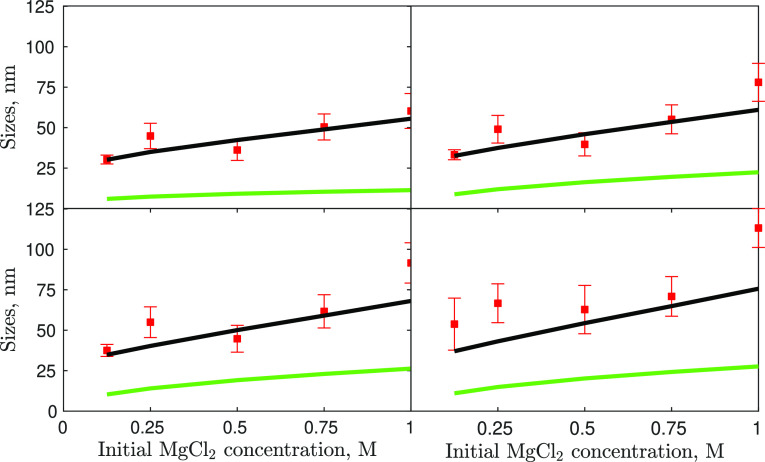
Characteristic sizes, from left to right and top to bottom, *d*_10_, *d*_21_, *d*_32_, *d*_43_, derived from the measured PSD (red symbols) and predicted by the model (black lines—all phenomena, green line—molecular processes only), versus the initial MgCl_2_ concentrations.

Figure 7 shows the comparison between the experimentally measured mean particle sizes at different initial reactant concentrations (cases #1–5 for tuning) and the model predictions using the inferred kinetics set.

**Figure 8:**
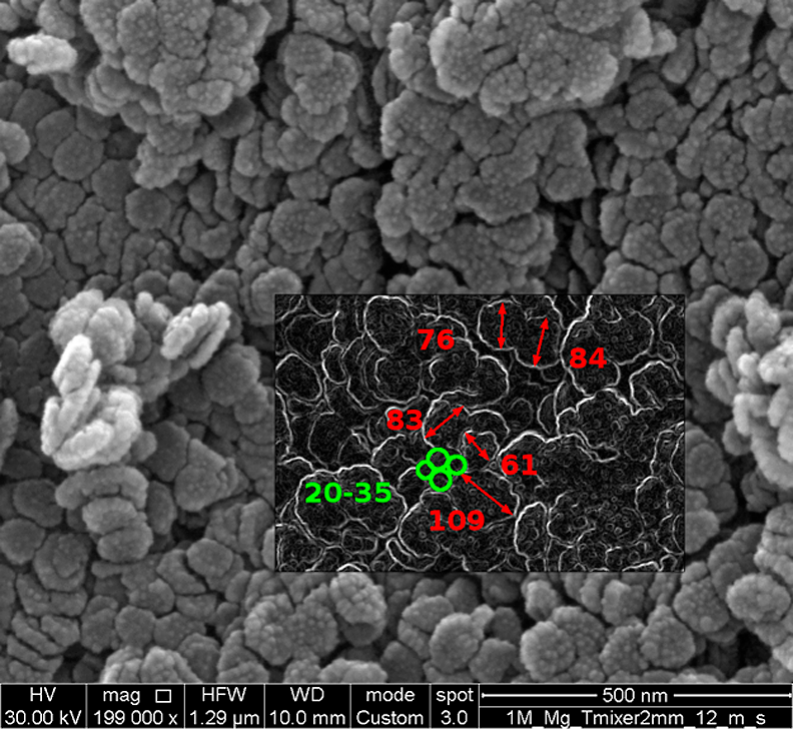
SEM analysis for case #5.

The black line refers to the predictions of the model when primary nucleation, growth, and aggregation are considered. The green line, on the other hand, represents the size that the primary particles would potentially have if their aggregation could be prevented. The comparison of the two trends, thus, reveals how important the contribution of aggregation is to the precipitation processes. For this reason, SEM analyses (see Figure 8) were performed to show how primary particles (green) of the order of magnitude of tens of nanometers cement to form larger aggregates (red) of hundreds of nanometers, in line with model predictions.

**Figure 9:**
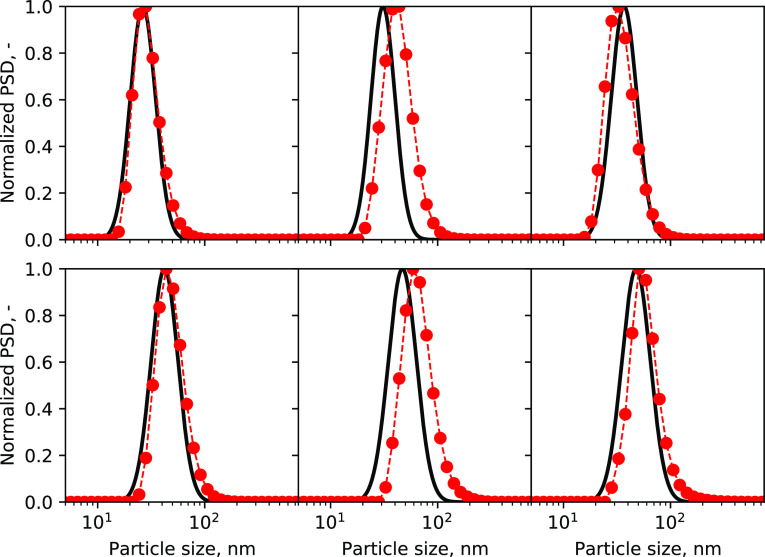
Reconstructed PSDs from their moments, compared with the experimental ones. Each comparison refers to the concentration set investigated #1, #2, #3, #4, #5, and #6. Black lines are the PSDs from simulations, whereas the red dotted lines are the PSDs from experimental tests (#1, top-left/#2, top-middle/#3, top-right/#4, bottom-left/#5, bottom-middle/#6, bottom-right).

The final parameters set provides good agreement with both the tuning dataset and the validation one. Concerning the tuning dataset, only two points (out of twenty) are slightly out of the standard deviations, and this might be due to a small morphology change^13^ not accounted for in the model. It is noteworthy that the *d*_43_ has a higher experimental uncertainty than the other characteristic sizes (*d*_10_, *d*_21_, and *d*_32_). This more pronounced deviation can be traced back to the difficulty of current experimental devices in correctly determining the tails of the distributions, especially when working in the nanometer range. In addition, it is noteworthy that the *d*_43_ is always underestimated. A possible explanation could lie in the simplified nature of the model. Indeed, the 1D model does not consider the spatial distribution (and radial dispersion) of the flow field. It should be considered that the velocity nulls at the wall and that turbulent energy dissipation increases. Particles near the wall, therefore, experience longer residence times and higher turbulence, which leads them to aggregate and, consequently, increase their size. In the distribution perspective, again, this effect is more pronounced for bigger particles (*d*_43_) and it mainly influences the tails. Despite underestimating all mean values of d_43_, the model still falls within the standard deviation. As expected, in the mixing channel the radial dispersion, although still present, is greatly reduced when the Reynolds number is sufficiently high. Moreover, the PSDs reconstructed from the moments by using the algorithm reported by John et al.,^34^ compare well with the PSDs measured at the T_2mm_-mixer outlet. Some PSDs are reported in Figure 9 for all the tuning dataset (cases #1–5) and for case #6 of the validation one.

The reconstruction algorithm takes as input the first three moments (and their ratios). In this perspective, therefore, a strong analogy can be found with the characteristic sizes in Figure 7. Reconstructed PSDs for cases #2 and #5 slightly deviate toward the left due to a small deviation in the relative *d*_10_, *d*_21_, and *d*_32_ computationally predicted. In general, a good agreement is obtained.

The identified kinetics set was further used to predict magnesium hydroxide PSDs at different operative conditions to assess the influence of mixing conditions and, thus, the need for a micro-mixing model. To do so, the validation set was employed. The effect of lower flow rates was investigated (i) reducing the flow rate in the mixing channel from 2320 mL/min (mean velocity in the mixing channel of 12.3 m/s, case #5) to 1602 (8.5 m/s, case #6) and 773 (4.1 m/s, case #7) mL/min, respectively; (ii) performing the Mg(OH)_2_ precipitation adopting a larger circular cross-shaped T_3mm_-mixer at a flow rate in the mixing channel of 2714 mL/min (mean velocity of 6.4 m/s, case #8). In all cases, 1 M MgCl_2_ and 2 M NaOH solutions were employed.

**Figure 10:**
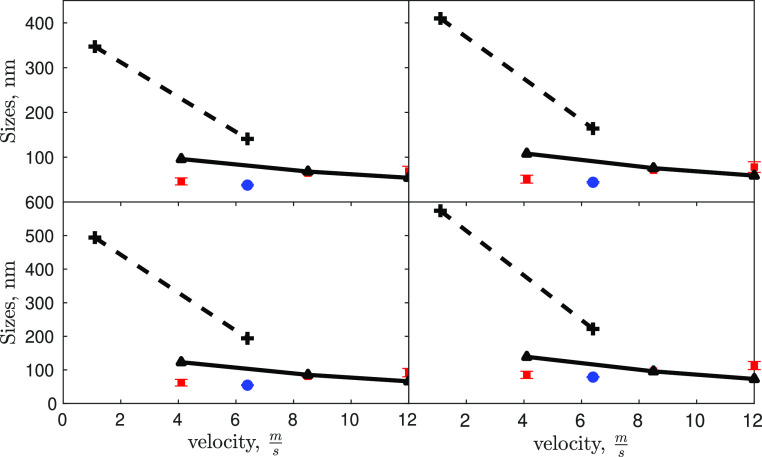
Characteristic sizes, from left to right and top to bottom, *d*_10_, *d*_21_, *d*_32_, *d*_43_, derived from the measured PSDs and predicted by the model at different flow rates or different mean velocities in the mixing channel. Effect of the velocity on the PSDs in two different systems. Experimental results in the T_2mm_-mixer (red squares) (i), experimental results in the T_3mm_-mixer (blue dot) (ii), simulations for the T_2mm_-mixer (solid line) (iii), and computational predictions for the T_3mm_-mixer (dashed line) (iv).

Figure 10 shows that for a mean velocity (i.e., flow rate) range in the mixing channel between 4 and 12 m/s, no significant changes in the mean particle size are detected. The model shows, however, a shallow increase in the mean particle size when the mean velocity in the mixing channel is reduced. This increase is more pronounced for mean velocity values smaller than 4 m/s. Validating this trend is complicated by several reasons. Performing experiments at flow rates smaller than 4 m/s is challenging but can be done for the T_3mm_-mixer (e.g., 207 mL/min corresponding to a velocity of 1.1 m/s in the mixing channel). Under these operating conditions, in fact, the developed measurement protocol cannot be employed, as this is valid only for particles smaller than 2000–3000 nm, but larger particles are instead observed. The Malvern Zetasizer Nano ZSP has an upper confidence limit, due to the sedimentation of the particles during measurements. This proves that larger particles are obtained by decreasing the flow rate. Measurements performed with another instrument (based on static light scattering) and another protocol indicate an approximate mean particle size ranging from 2000 to 4000 nm. However, this data point is not added to Figure 10 as it is not comparable with the other experimental data points. Another issue is related to the fact that by decreasing the mean velocity the turbulence levels in the T-mixer decrease and the employed turbulence model is affected by larger uncertainties. For these reasons, it is not possible to make definitive conclusions on the ability of the model to describe these effects. These latter are indeed the subject of our future work, performed on a mixer and on an experimental rig that allows for these investigations. At last, it is also worth mentioning that for case #8 in the T_3mm_-mixer, model predictions overestimate the particle sizes. A possible reason can lie in the nature of the produced particles. As reported in Figure 5 in Battaglia et al.,^17^ magnesium hydroxide volume-based PSDs, measured with static light scattering, clearly show a bimodal distribution of nano-sized and micron-sized aggregates. This can cause an under-estimation of particle size, when a Malvern Zetasizer Nano ZSP is adopted, since the Brownian motion of small particles is higher than that of big ones. These considerations can justify the model and experimental discrepancy evaluated in the case of the T_3mm_-mixer.

In the end, a physical interpretation of the obtained kinetics parameters is provided. Homogeneous nucleation parameters lead to the final total particles number density of approximately 10^18^ particles no./m^3^ in line with what is expected for sparingly soluble salts.^35^ Heterogeneous nucleation parameters reflect what is known in the literature. In this regard, its rate is supposed to have a lower slope, which is translated into a lower exponential factor,^22^ (i.e., the lower interfacial tension between the nucleation sites and the solution) and the pre-exponential factor to be orders of magnitude lower.

**Figure 11:**
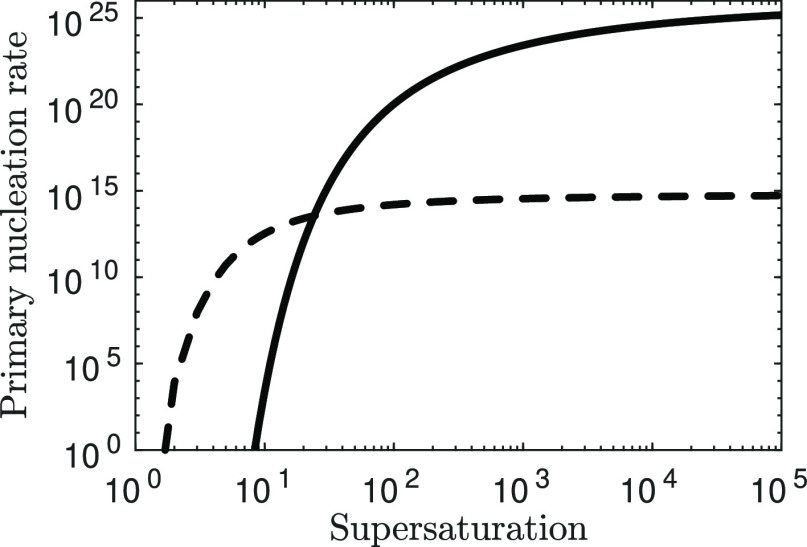
Homogeneous nucleation rate (solid line) compared with the heterogeneous one (dashed line) using the inferred primary nucleation parameters.

Figure 11 shows the homogeneous and heterogeneous nucleation rates (particles no./m^3^/s) for different supersaturation values. For high supersaturation values (left-hand side of Figure 11) homogeneous nucleation is much higher than the heterogeneous one, whereas for low supersaturation values (right-hand side of Figure 11) heterogeneous nucleation becomes more relevant compared to the homogeneous one. Therefore, considering only one of the two might lead to significant errors. Regarding the growth rate equation, the same expression was found in Alamdari et al.^15^

## 5 Conclusions

In the present work, a comprehensive study for the magnesium hydroxide precipitation kinetics was proposed. It was focused experimentally on the physical characterization of the suspension downstream of two T-mixers and mathematically on building up a model exploiting PFR fluid-dynamics hypothesis to infer the process precipitation kinetics. The best fitting parameters set was derived from the constrained optimization to avoid unphysical model behavior using the algorithm in the MatLab environment. The inferred kinetics set was validated by exploiting a second experimental dataset at different operating conditions. Experimental PSDs were compared with those provided by the model, exploiting a reconstruction algorithm that starts from the moments. In these operating conditions, primary nucleation and aggregation can be considered the dominant precipitation phenomena due to the very fast micro-mixing that leads to massive local supersaturation, and then to very fast aggregation because of the suspension instability due to the low Zeta potential. The proposed framework, therefore, accounts for all the main involved phenomena and for the first-time kinetics parameters are proposed for magnesium hydroxide precipitation. Moreover, the micro-mixing model plays a critical role in accurately describing the experimental data. Without it, the model would fail to describe the experimental data, and the results would be unphysical since the kinetics set would be affected by the mixing effect. The proposed framework aims to provide a set of kinetics parameters that are independent of the operative conditions and system geometry. Ideally, this framework should accurately describe the experimental data regardless of changes in these variables. While the model is successful in achieving this goal to a large extent, it cannot perfectly predict the experimental data when changes occur. For instance, the model cannot accurately predict the experimental data when the flow rate is changed within the same T-mixer or when a new T-mixer with a larger diameter is employed. This discrepancy can be attributed to both the model and the experimental uncertainty, which cannot be fully eliminated. Therefore, it is important to consider potential experimental variability when interpreting the model’s results. Nonetheless, this study provides a solid foundation for future research and development, and the proposed framework will be a valuable tool for designing first-generation prototypes. Additionally, future investigations into secondary nucleation mechanisms, not considered in this study due to their irrelevance to the T-mixer system, will be necessary to further our understanding of magnesium hydroxide precipitation kinetics.

## Supporting Information Available

Additional details regarding the β-PDF approach to integrate the mixing effect into the precipitation process; details regarding the solution activity coefficient calculation; simulation results and discussion when micro-mixing is not considered; and considerations regarding the CFD modeling (PDF)
