# Supplementary material for: Computational Modeling of Magnesium Hydroxide Precipitation and Kinetics Parameters Identification
Source: Cryst Growth Des. 2023 Jun 23;23(7):4748–59. doi: 10.1021/acs.cgd.2c01179 (PMC10327471; doi:10.1021/acs.cgd.2c01179)
Supplement: Supplementary file 1 — cg2c01179_si_001.pdf [file cg2c01179_si_001.pdf]

Supporting information for “Computational modelling of Magnesium Hydroxide precipitation and kinetics parameters identification”

AUTHOR NAMES

Antonello Raponi[1], Salvatore Romano[2], Giuseppe Battaglia[2], Antonio Buffo[1], Marco Vanni[1], Andrea Cipollina[2], Daniele Marchisio[1]

AUTHOR ADDRESS

[1] Department of Applied Science and Technology, Institute of Chemical Engineering - Politecnico di Torino, Torino, 10129, Italy

[2] Dipartimento di Ingegneria, Università degli Studi di Palermo - viale delle Scienze Ed.6, 90128 Palermo, Italy

KEYWORDS.
Precipitation; Magnesium Hydroxide; Dynamic Light Scattering; Computational Modelling; QMOM

β-PDF approach

The chemical reaction between magnesium and hydroxyl ions is instantaneous and irreversible and algebraic equations can be used to model it. Mixture fraction and its variance are enough to determine effective ion concentrations that can precipitate in crystal form. Being c_A, c_B the components concentrations depending on the local mixture fraction (α), ${c_A}^0, {c_B}^0$ the initial concentrations and α_s the stoichiometric mixture fraction, the following equations can be written for $A + rB \rightarrow P$ reaction:

$$\begin{cases} \dfrac{c_A}{{c_A}^0} = \left(1 - \dfrac{\alpha}{\alpha_s}\right) \\ c_A = 0 \end{cases} \qquad \begin{cases} c_B = 0 \\ \dfrac{c_B}{{c_B}^0} = \left(\dfrac{\alpha - \alpha_s}{1 - \alpha_s}\right) \end{cases} \qquad \begin{cases} c_P = {c_B}^0 \alpha & 0 \le \alpha \le \alpha_s \\ c_P = {c_B}^0 \alpha_s \left(\dfrac{\alpha - 1}{\alpha_s - 1}\right) & \alpha_s \le \alpha \le 1 \end{cases} \qquad (1)$$

$$\alpha_s = \frac{r{c_A}^0}{r{c_A}^0 + {c_B}^0}$$

As provided by these equations, since chemical reaction is instantaneous, reactants can’t coexist when they are molecularly micro-mixed. Depending on fluid-dynamics conditions, though, reaction could be stunted. Based on the variance value, a probability density function is used to determine the probability that reactants are micro-mixed or micro-segregated in a volume of

fluid. In other words, if variance is high enough (micro-segregated system), reaction doesn't begin depending on the lack of molecularly micro-mixed reactants and only reactants dilution arises.

$$\beta = \frac{\alpha^{\nu-1}(1-\alpha)^{w-1}}{\int_0^1 \alpha^{\nu-1}(1-\alpha)^{w-1}} \qquad (2)$$

$$\nu = \bar{\alpha}\left(\frac{\bar{\alpha}(1-\bar{\alpha})-\bar{\alpha}'^2}{\bar{\alpha}'^2}\right), w = (1-\bar{\alpha})\left(\frac{\bar{\alpha}(1-\bar{\alpha})-\bar{\alpha}'^2}{\bar{\alpha}'^2}\right)$$

For the 1D model, mixture fraction is kept constant and equal to the stoichiometric mixture fraction value in line with experiments. The stoichiometric mixture fraction value represents, in the mixture fraction space, the value at which reactants are stoichiometrically reacting. Variance time evolution and mixture fraction knowledge led to products concentrations calculation.

$$\overline{c_A} = \int_0^1 (c_A\beta)(\alpha)\mathrm{d}\alpha$$

$$\overline{c_B} = \int_0^1 (c_B\beta)(\alpha)\mathrm{d}\alpha \qquad (3\text{-a,b,c})$$

$$\overline{c_P} = \int_0^1 (c_P\beta)(\alpha)\mathrm{d}\alpha$$

Full derivation is reported in sec. *Analytical derivation*. Authors aim is to give, for the first time concerning magnesium hydroxide precipitation, a comprehensive description of the fluid-dynamics interference on the molecular precipitation phenomena (i.e., primary nucleation and growth). Assuming an initial concentration of magnesium chloride equal to 1 M (for the sake of clarity), equation 3-c can be properly written as:

$$\overline{c_P} = \int_0^1 (c_P\beta)(\alpha)d\alpha = c_B(t)f(\nu,w) = c_B(t)f\left(\bar{\alpha},\bar{\alpha}'^2\right) \qquad (4)$$

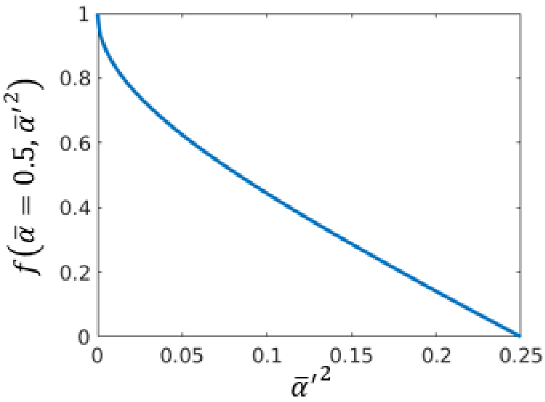

Figure S1. Micro-mixing effect on the molecular processes (i.e., primary nucleation and growth) quantified through the $\beta - PDF$ approach

Rearranging properly eq. (4), $f\left(\bar{\alpha}, \bar{\alpha}'^2\right)$ comes out and can be interpreted physically, having in mind the micro-mixing effect. At the very beginning of the simulation, variance is the highest possible; it means that $f\left(\bar{\alpha}, \bar{\alpha}'^2\right) \to 0$. At the beginning, due to the micro-segregation of the components, no molecular process can begin, which results in zero micro-mixing. On the other hand, as soon as the turbulence increases, variance is dissipated by turbulent eddies and reactants can interact within the Batchelor scale. The variance tends to zero, as described in Figure 5 (manuscript), and $f\left(\bar{\alpha}, \bar{\alpha}'^2\right) \to 1$ (Figure S1). It means that, due to turbulence, the free-stream ions available can now integrally react.

Analytical derivation

$$\overline{c_A} = \int_0^1 (c_A\beta)(\alpha)d\alpha$$

$$\overline{c_B} = \int_0^1 (c_B\beta)(\alpha)d\alpha$$

$$\overline{c_P} = \int_0^1 (c_P\beta)(\alpha)d\alpha$$

These three integrals are closed analytically; all three derivations are reported in this Appendix. Not to weight down the notation, let's denote

$$\int_0^1 \alpha^{\nu-1}(1-\alpha)^{w-1} = B(\nu, w) = \frac{\Gamma(\nu)\Gamma(w)}{\Gamma(\nu+w)}$$
$$\int_0^1 \alpha^{\nu}(1-\alpha)^{w-1} = B(\nu+1, w) = \frac{\Gamma(\nu+1)\Gamma(w)}{\Gamma(\nu+w+1)}$$
$$\frac{1}{B(\nu,w)}\int_0^{\alpha_s} \alpha^{\nu-1}(1-\alpha)^{w-1} = I(\nu, w)$$
$$\frac{1}{B(\nu+1,w)}\int_0^{\alpha_s} \alpha^{\nu}(1-\alpha)^{w-1} = I(\nu+1, w)$$

where Γ is the gamma function.

$$\overline{c_A} = \int_0^1 (c_A\beta)(\alpha)d\alpha = \int_0^{\alpha_s} {c_A}^0\left(1-\frac{\alpha}{\alpha_s}\right)\frac{\alpha^{\nu-1}(1-\alpha)^{w-1}}{B(\nu,w)}d\alpha$$
$$= \frac{{c_A}^0}{B(\nu,w)\alpha_s}\int_0^{\alpha_s}(\alpha_s-\alpha)\alpha^{\nu-1}(1-\alpha)^{w-1}d\alpha$$
$$= \frac{{c_A}^0}{B(\nu,w)\alpha_s}\left[\alpha_s\int_0^{\alpha_s}\alpha^{\nu-1}(1-\alpha)^{w-1}d\alpha - \int_0^{\alpha_s}\alpha^{\nu}(1-\alpha)^{w-1}d\alpha\right]$$
$$= \frac{{c_A}^0}{B(\nu,w)\alpha_s}\left[\alpha_s I(\nu,w)B(\nu,w) - I(\nu+1,w)B(\nu+1,w)\right]$$

For B component:

$$\overline{c_B} = \int_0^1 (c_B\beta)(\alpha)d\alpha = \int_{\alpha_s}^1 c_B{}^0 \left(\frac{\alpha - \alpha_s}{1 - \alpha_s}\right) \frac{\alpha^{\nu-1}(1-\alpha)^{w-1}}{B(\nu, w)} d\alpha$$
$$= \frac{c_B{}^0}{B(\nu, w)(1 - \alpha_s)} \int_{\alpha_s}^1 (\alpha - \alpha_s)\alpha^{\nu-1}(1-\alpha)^{w-1} d\alpha$$
$$= \frac{c_B{}^0}{B(\nu, w)(1 - \alpha_s)} \left[\int_{\alpha_s}^1 \alpha^{\nu}(1-\alpha)^{w-1} d\alpha - \int_{\alpha_s}^1 \alpha_s \alpha^{\nu-1}(1-\alpha)^{w-1} d\alpha\right]$$
$$= \frac{c_B{}^0}{B(\nu, w)(1 - \alpha_s)} \left[B(\nu+1, w)\big(1 - I(\nu+1, w)\big) - \alpha_s B(\nu, w)\big(1 - I(\nu, w)\big)\right]$$

For the P component:

$$\overline{c_P} = \int_0^1 (c_P\beta)(\alpha)d\alpha$$
$$= \int_0^{\alpha_s} c_B{}^0 \alpha \frac{\alpha^{\nu-1}(1-\alpha)^{w-1}}{B(\nu, w)} d\alpha + \int_{\alpha_s}^1 c_B{}^0 \alpha_s \left(\frac{\alpha - 1}{\alpha_s - 1}\right) \frac{\alpha^{\nu-1}(1-\alpha)^{w-1}}{B(\nu, w)} d\alpha$$
$$= \frac{c_B{}^0}{B(\nu, w)} \int_0^{\alpha_s} \alpha^{\nu}(1-\alpha)^{w-1} d\alpha + \frac{c_B{}^0 \alpha_s}{B(\nu, w)(\alpha_s - 1)} \int_{\alpha_s}^1 (\alpha - 1)\alpha^{\nu-1}(1-\alpha)^{w-1} d\alpha$$
$$= \frac{c_B{}^0}{B(\nu, w)} \int_0^{\alpha_s} \alpha^{\nu}(1-\alpha)^{w-1} d\alpha + \frac{c_B{}^0 \alpha_s}{B(\nu, w)(\alpha_s - 1)} \left[\int_{\alpha_s}^1 \alpha^{\nu}(1-\alpha)^{w-1} d\alpha - \int_{\alpha_s}^1 \alpha^{\nu-1}(1-\alpha)^{w-1} d\alpha\right]$$
$$= \frac{c_B{}^0}{B(\nu, w)} I(\nu+1, w) B(\nu+1, w) + \frac{c_B{}^0 \alpha_s}{B(\nu, w)(\alpha_s - 1)} \left[B(\nu+1, w)\big(1 - I(\nu+1, w)\big) - B(\nu, w)\big(1 - I(\nu, w)\big)\right]$$

Bromley's activity coefficient

At the beginning of activity calculation for a solute in multi-component solution indexes must be fixed and used for related equation. In this regard, since Mg^{+2} and OH^- ions are needed for supersaturation, following indexes were given:

Mg^{+2} index: 1 Na^+ index: 3

OH^- index: 2 Cl^- index: 4

Thus, following the above-mentioned steps, multi-component solution parameters were calculated using these equations:

$$F_1 = Y_{21} \log \gamma_{12}^0 + Y_{41} \log \gamma_{14}^0 + \frac{A_\gamma I^{0.5}}{1 + I^{0.5}} [Z_1 Z_2 Y_{21} + Z_1 Z_4 Y_{41}] \quad (5)$$

$$F_2 = X_{12} \log \gamma_{12}^0 + X_{32} \log \gamma_{32}^0 + \frac{A_\gamma I^{0.5}}{1 + I^{0.5}} [Z_1 Z_2 X_{12} + Z_3 Z_2 X_{32}] \quad (6)$$

where odd indexes refer to cations and even ones to anions, $A_\gamma = 0.511 \sqrt{\frac{kg}{mol}}$ and I is the solution ionic strength calculated as:

$$I = \frac{1}{2} \sum_i c_i Z_i^2 \quad (7)$$

where c_i is the concentration of the ith ions in the solution. Moreover, parameters needed have this form:

$$Y_{i1} = \left(\frac{Z_i + Z_1}{2}\right)^2 \frac{m_i}{I} \quad (8)$$

$$X_{j2} = \left(\frac{Z_2 + Z_j}{2}\right)^2 \frac{m_j}{I} \quad (9)$$

where m_i is the ith ions molality and $\log \gamma_{ij}^0$ is the ten-based logarithm of activity coefficient of the pseudo-solution with the same ionic strength of the multi-component one but considering only the *i-j* ions pair. The governing equation is:

$$\log \gamma_{ij}^0 = \frac{-A_\gamma |Z_i Z_j| I^{0.5}}{1 + I^{0.5}} + \frac{(0.06 + 0.6B)|Z_i Z_j| I}{\left(1 + \frac{1.5}{|Z_i Z_j|} I\right)^2} + BI \quad (10)$$

where B is an empirical parameter which can be calculated or found in the literature [1]. For $MgCl_2$, NaOH and NaCl B values from the table were used while for $Mg(OH)_2$ B value was estimated using Bromley correlation:

$$B = B_{cation} + B_{anion} + \delta_{cation} \delta_{cation} \quad (11)$$

where B_{cation}, B_{anion}, δ_{cation}, δ_{anion} are tabled in literature. Eventually, concluding with the three steps list, it is possible to evaluate the $\log \gamma_{12}$ for Mg^{+2} – OH^- in the considered multi-component solution with the equation:

$$\log \gamma_\pm = \frac{-A_\gamma \left(\frac{\sum_i \nu_i Z_i^2}{\nu}\right) I^{0.5}}{1 + I^{0.5}} + \frac{1}{\nu} \sum_i \nu_i F_i \quad (12)$$

where $\nu = \sum_i \nu_i$. This general form is simplified because the i index refers only to the ions generated by the salt for which activity is required; in this case, i=1,2 refers to Mg^{+2} and OH^{-} ions. Therefore, in the present work, it results in the equation:

$$\log \gamma_{\pm} = \frac{-A_{\gamma}\left(\frac{\nu_1 Z_1^2 + \nu_2 Z_2^2}{\nu_1 + \nu_2}\right) I^{0.5}}{1 + I^{0.5}} + \frac{1}{\nu}(\nu_1 F_1 + \nu_2 F_2) \quad (13)$$

Micro-mixing modelling

As a test, we turned off the variance calculation in our model, effectively assuming instantaneous mixing and precipitation upon entering the T-mixers. However, the predictions for mean particle sizes based on this assumption were clearly off, as shown in Figure S2.

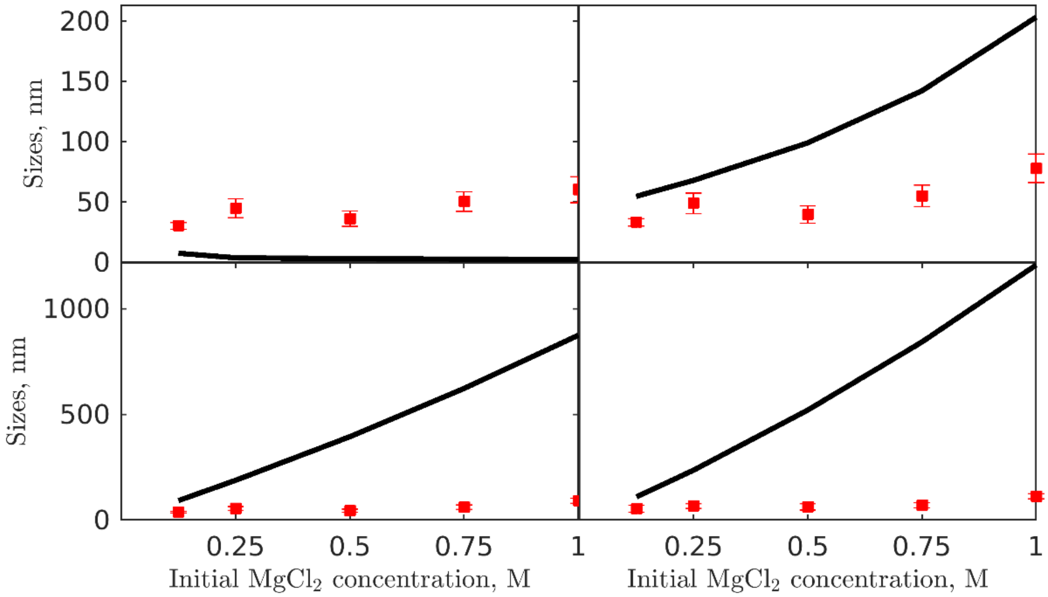

Figure S2. Characteristic sizes, from left to right and top to bottom, d_{10}, d_{21}, d_{32}, d_{43}, derived from the measured PSD and predicted by the model. Comparison between model's predictions using the inferred kinetics parameters set (manuscript), deactivating the micro-mixing model, and the experimental data (Cases #1-5).

The predictions failed to capture the correct values and trends observed in experimental data. We further understood the importance of the micro-mixing model by comparing the supersaturation evolution predicted by the model both with and without the micro-mixing, as shown in Figure S3. Only when accounting for the micro-mixing (Figure S3, top) the correct trend was observed, with supersaturation first generated by mixing and then consumed by precipitation. On the other hand, when micro-mixing was neglected (Figure S3, bottom), precipitation started immediately, resulting in larger supersaturations.

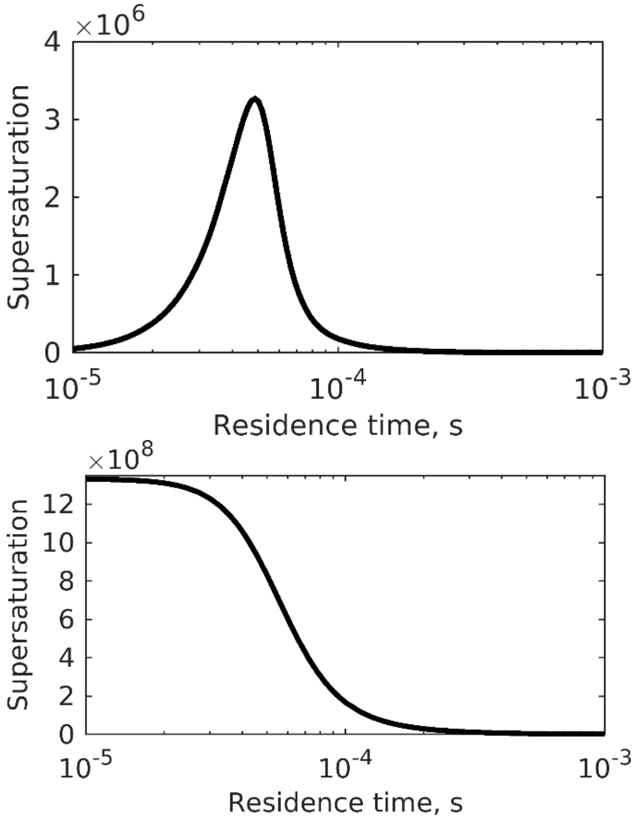

Figure S3. Supersaturation profile obtained by employing the micro-mixing model (top). Supersaturation profile obtained without employing the micro-mixing model (bottom).

We also optimized the model parameters by fitting the experimental data, but this time without the micro-mixing model. Despite having the same number of parameters as the full model, the resulting parameter set was unable to reproduce the experimental trends shown in Figure S4 (cases #1-5, top; cases #6-8, bottom).

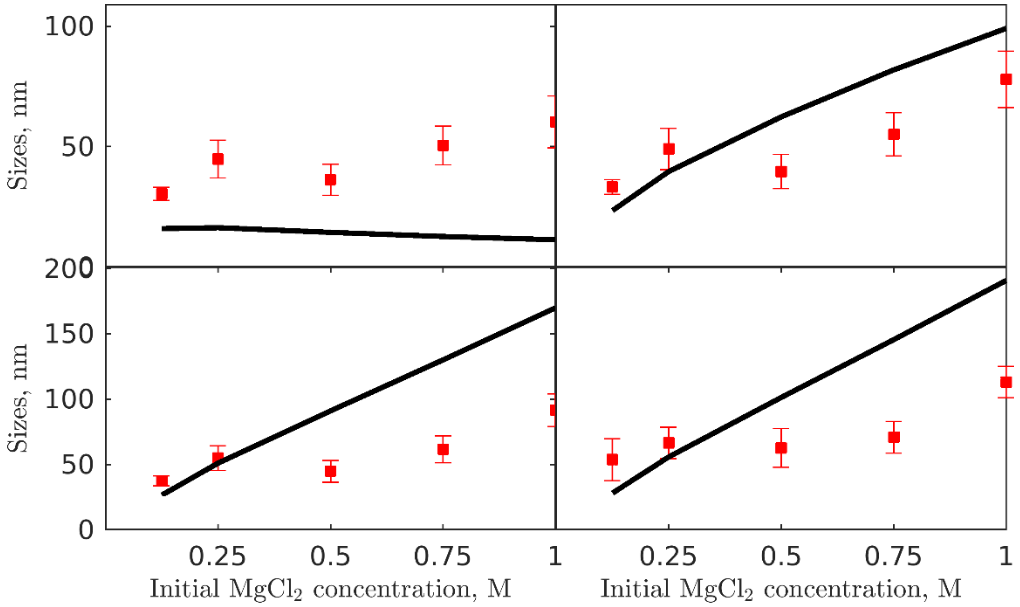

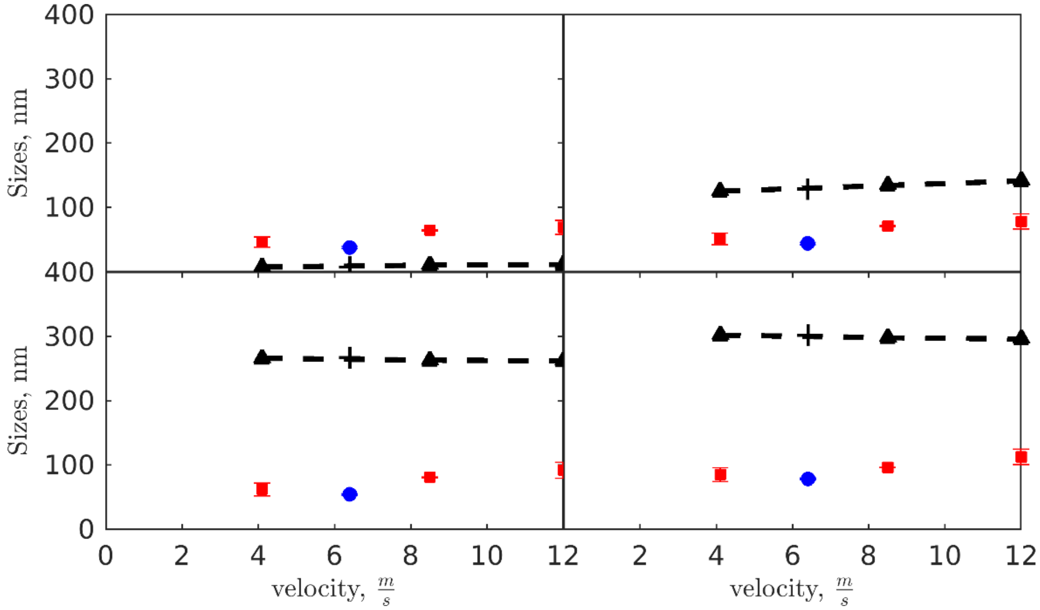

Figure S4. Characteristic sizes, from left to right and top to bottom, d_{10}, d_{21}, d_{32}, d_{43}, derived from the measured PSD and predicted by the model. Comparison between model (without micro-mixing) outcome and experimental data used for fitting (cases #1-5, top). Comparison between model predictions and experimental data used for testing (cases #6-8, bottom). Effect of velocity on the PSDs in two different systems. Experimental results in the T_{2mm}-mixer (red squares) (i), experimental results in the T_{3mm}-mixer (blue dot) (ii), simulations for the T_{2mm}-mixer (dashed line) (iii), computational prediction for the T_{3mm}-mixer (cross marker) (iv)

We observed significant differences in all the parameters (Table S1), especially A_1, which is involved in the homogeneous nucleation rate. The fact that A_1 decreased by three orders of magnitude, roughly corresponding to the increase in supersaturation shown in Figure S3, highlights the crucial role of micro-mixing in our description.

	A_1	A_2	B_1	B_2	k_g	G	C_1	A_p
Value	23	15	315	50	-12.2	1.1	4.4	2.67

Table S1. The optimal set of parameters obtained from the comparison between the model without micro-mixing and experimental data (cases #1-5).

CFD component

CFD simulations might be used to estimate the mixing time and the Kolmogorov timescale, which are important parameters in predicting the particle size distribution.

$$u\frac{\mathrm{d}\bar{\alpha}'^2}{\mathrm{d}y} = -\frac{C_\phi}{2}\frac{\varepsilon(y)}{k(y)}\bar{\alpha}'^2 \tag{15}$$

$$\beta_{ij}^{(turb)} = \sqrt{\frac{8\pi}{15}}\sqrt{\frac{\varepsilon}{\nu}}\left(\frac{L_i + L_j}{2}\right)^3 \tag{16}$$

The mixing time is the time required for two fluids to mix completely, and it is proportional to the ratio between the turbulent kinetic energy (k) and the turbulent dissipation rate (ε) (Eq. (15)).

The Kolmogorov timescale is the smallest timescale at which energy is dissipated in a turbulent flow, and it is proportional to the square root of the kinematic viscosity (ν) divided by the turbulent dissipation rate (Eq. (16)). These two parameters affect the degree of mixing, which in turn affects all the precipitation phenomena, and therefore, the resulting particle size distribution. While it is true that mixing time can be estimated using empirical correlations, such as those discussed in our previous work[2], the accuracy of the estimates for the ε value may not always be high. For instance, it could be possible to estimate the ε value by passing through the pressure drops. Nevertheless, empirical correlations would not provide a detailed estimation of pressure drops, especially those due to the impingement between fluids. In this case an underestimate of the ε value, could lead to a quite important deviation of the model predictions as shown in Figure S5.

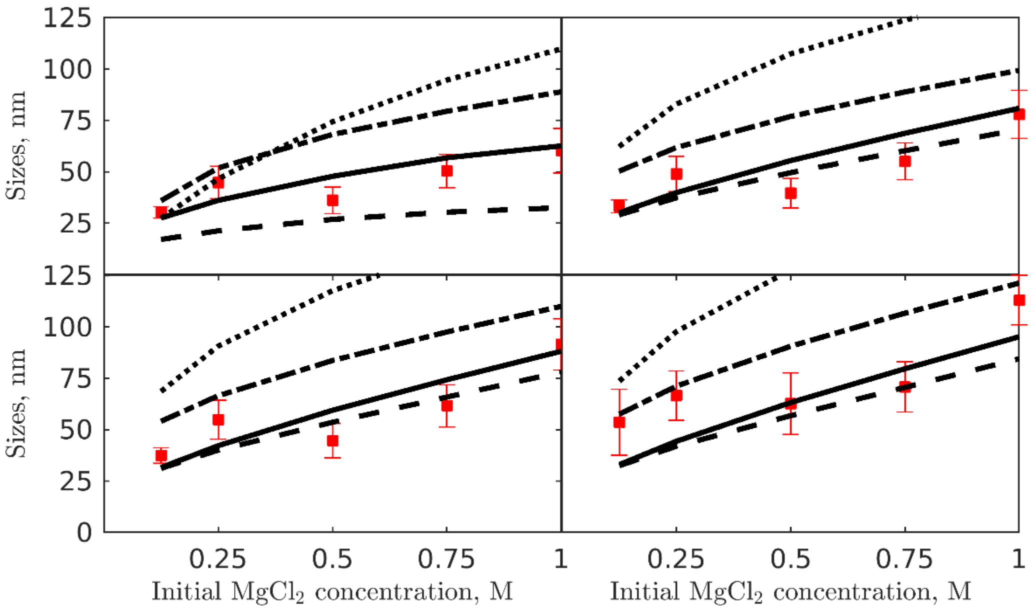

Figure S5. Characteristic sizes, from left to right and top to bottom, d_{10}, d_{21}, d_{32}, d_{43}, derived from the measured PSD and predicted by the model. Model predictions at different values (constants) of ε. The solid line refers to the model with the integration of the CFD component. The dashed line refers to the model with constant ε value of 10^5 m^2/s^3. The dash-dotted line refers to the model with constant ε value of 10^3 m^2/s^3. The dotted line refers to the model with constant ε value of 10 m^2/s^3.

On the other hand, CFD simulations provide a more comprehensive understanding of the mixing process and can account for the effect of various parameters, such as fluid properties, mixer geometry, and flow rates, on the turbulence characteristics. Therefore, although other methods may be used to estimate the mixing time and turbulence parameters, CFD simulations remain a valuable tool for obtaining reliable and comprehensive information on the mixing process [3,4].

References

(1) Bromley, L. A. Thermodynamic Properties of Strong Electrolytes in Aqueous Solutions. *AIChE Journal* **1973**, *19* (2), 313–320. https://doi.org/10.1002/aic.690190216.

(2) Battaglia, G.; Romano, S.; Raponi, A.; Marchisio, D.; Ciofalo, M.; Tamburini, A.; Cipollina, A.; Micale, G. Analysis of Particles Size Distributions in Mg(OH)2 Precipitation from Highly Concentrated MgCl2 Solutions. *Powder Technol* **2022**, *398* (Article 117106), 1–10. https://doi.org/10.1016/j.powtec.2021.117106.

(3) Para, M. L.; Alidoost, M.; Shiea, M.; Boccardo, G.; Buffo, A.; Barresi, A. A.; Marchisio, D. A Modelling and Experimental Study on the Co-Precipitation of Ni0.8Mn0.1Co0.1(OH)2 as Precursor for Battery Cathodes. *Chem Eng Sci* **2022**, *254*, 117634. https://doi.org/10.1016/j.ces.2022.117634.

(4) Orlewski, P. M.; Mazzotti, M. Modeling of Mixing-Precipitation Processes: Agglomeration. *Chem Eng Technol* **2020**, *43* (6), 1029–1039. https://doi.org/10.1002/ceat.201900551.
